# Rasd1 interacts with Ear2 (Nr2f6) to regulate renin transcription

**DOI:** 10.1186/1471-2199-12-4

**Published:** 2011-01-19

**Authors:** Jen Jen Tan, Shufen Angeline Ong, Ken-Shiung Chen

**Affiliations:** 1 School of Biological Sciences, Department of Genomics and Genetics, Nanyang Technological University, 60 Nanyang Drive, 637551, Singapore; 2 Agency for Science, Technology and Research, 1 Fusionpolis Way, #20-10 Connexis North Tower, 138632, Singapore

## Abstract

**Background:**

The Rasd1 protein is a dexamethasone induced monomeric Ras-like G protein that oscillates in the suprachiasmatic nucleus (SCN). Previous studies have shown that Rasd1 modulates multiple signaling cascades. However, it is still unclear exactly how Rasd1 carries out its function. Studying protein-protein interactions involving Rasd1 may provide insights into its biological functions in different contexts.

**Results:**

To further explore the molecular function of Rasd1, we performed a yeast two-hybrid screen and identified Ear2, a negative regulator of renin transcription, as an interaction partner of Rasd1. We validated the interaction *in vitro *and in transfected COS-7 cells. We further confirmed the interaction of endogenous Rasd1 and Ear2 from HEK293T cell and mouse brain extract. Rasd1 inhibited transcriptional repression by Ear2 on a renin promoter-luciferase reporter construct both in the presence and absence of all-trans-retinoic acid. Moreover, real-time RT-PCR showed upregulation of endogenous renin transcription in As4.1 cells over-expressing Rasd1. We demonstrated that the ligand binding domain of Ear2 is required for physical and functional interaction between the two proteins. In addition, we demonstrated that shRNA-mediated knockdown of Rasd1 results in further repression of Ear2-mediated renin transcription, whereas induction of Rasd1 by dexamethasone counteracts the effects of shRNA-mediated Rasd1 knockdown. Finally, our study showed that Rasd1 missense mutations not only attenuate their physical interaction with Ear2 but also abolish their ability to counteract repression of renin transcription mediated by Ear2.

**Conclusions:**

Our study provides evidence for physical and functional interactions between Rasd1 and Ear2. The results suggest that their interactions are involved in renin transcriptional regulation. These findings not only reveal a novel role for Rasd1-medated signaling but also provide the basis for potential intervention of renin expression.

## Background

The renin-angiotensin system plays a major physiological role in the control of blood pressure, fluid volume and electrolyte balance. A functional renin-angiotensin system is also essential for the normal development of the mammalian renal system [1]. Renin, an aspartyl protease, is the rate-limiting enzyme in the renin-angiotensin enzymatic cascade which leads to the production of angiotensin II (Ang II), a vasoactive peptide and major effector molecule in the renin-angiotensin system [2,3]. Renin gene expression is largely regulated at the transcriptional level, although post transcriptional regulation has also been reported [4,5]. A potent classical transcriptional enhancer was identified ~2.6 kb upstream of the mouse renin gene, and this enhancer is homologous to a sequence ~12 kb upstream of the human renin gene [6-8]. The transcriptional enhancer contains several transcription factor binding sites that have both excitatory and inhibitory regulatory functions [9-12].

One protein that has been identified to bind to and regulate the renin enhancer is Ear2 [13]. It was determined that Ear2 negatively regulates renin expression by competing with retinoic acid receptor/ retinoid X receptor (RAR/RXR) for binding to the retinoic acid response elements (RARE) on the renin enhancer [13]. Ear2 is an orphan nuclear hormone receptor that belongs to the chicken ovalbumin upstream promoter-transcription factors (COUP-TF) gene family [14]. COUP-TFs have been shown to bind to a number of variable direct and indirect repeats with different spacings between the repeats [15] to affect a large plethora of genes [16-19]. COUP-TFs have been proposed to inhibit transactivation of other nuclear receptors through multiple mechanisms, including competitively binding to regulatory elements, competitively binding to RXR, mediating active repression via direct binding to regulatory elements, and mediating transrepression of another nuclear receptor without binding to DNA itself [14,20-22]. Nuclear hormone receptors have the ability to bind directly to DNA and regulate the expression of specific target genes; therefore, they are extremely crucial to the development, homeostasis and metabolism of an organism [16,20,21,23-25]. *Ear2 *is expressed in tissues of all major systems, and its expression has been implicated in the regulation of gene expression for normal embryo development [26,27]. Ear2 knockout mice are viable and fertile, but they possess circadian and nociception defects and abnormal locus coeruleus (LC) development [25].

Using a yeast two-hybrid system, we identified Ear2 as an interacting protein of Rasd1. Rasd1 is a G-protein that belongs to the RAS superfamily of small GTPases. It was first discovered as a dexamethasone-inducible gene in the AtT-20 pituitary cell line [28]. *Rasd1 *is an oscillating gene specific to the suprachiasmatic nucleus (SCN) [29]. Rasd1 modulates several signaling cascades and has several known functions. It interacts with neuronal nitric oxide synthase via CAPON to enhance physiological nitric oxide signaling [30]. Rasd1 is also crucial in the regulation of the responsiveness of the circadian clock to external stimuli [31]. In this study, we demonstrate that the interaction between Rasd1 and Ear2 regulates renin transcription. Our findings reveal a novel regulatory role of Rasd1 and a novel regulatory mechanism for Ear2-mediated renin transcription.

## Results

*Identification of Ear2 as a Rasd1 interacting protein*- To identify the proteins that are associated with Rasd1, we conducted a yeast two-hybrid screen. A mouse brain cDNA library was screened against full length Rasd1. All surviving colonies after nutritional selection were screened for *LacZ *expression using β-galactosidase. Table 1 lists the proteins that were identified. Ear2 is the protein for which the highest number of independent clones was obtained, with a total of three clones identified. To confirm the specificity of interaction between Rasd1 and Ear2, a separate yeast two-hybrid analysis was performed by mating yeast containing the Ear2 cDNA plasmid with yeast that already carried *Rasd1*. This was followed by nutritional selection and β-galactosidase assay. Ear2 again showed positive interactions with Rasd1 (not shown).

**Table 1:** Proteins identified from the yeast two-hybrid screen

No	Protein/Gene	Gene Name	Genbank Accession No.
1	Cenpb	Mus musculus centromere protein B	NM_007682.2

2	Gnb1	Mus musculus guanine nucleotide binding protein, beta 1	NM_008142.3

3	Nr2f6	Mus musculus nuclear receptor subfamily 2, group F, member 6	NM_010150.2

4	Plscr1	Mus musculus phospholipid scramblase 1	NM_011636.2

5	Sh3gl2	Mus musculus SH3-domain GRB2-like 2	NM_019535.2

6	Supt16 h	Mus musculus suppressor of Ty 16 homolog	NM_033618.2

7	Trp53bp2	Mus musculus transformation related protein 53 binding protein 2	NM_173378.2

8	Ywhah	Mus musculus tyrosine 3-monooxygenase/tryptophan 5-monooxygenase activation protein, eta polypeptide	NM_011738.1

**Figure 1:**
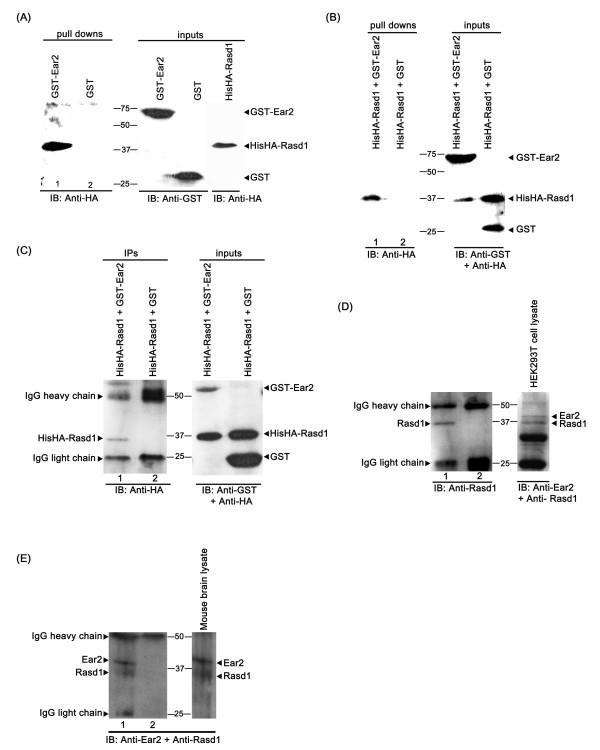
**Rasd1 and Ear2 interact *in vitro *and in living cells**. (A) *In vitro *binding of GST-Ear2 and HisHA-Rasd1. COS-7 cells were transfected with plasmids expressing the indicated constructs. Cell lysates containing HisHA-Rasd1 was incubated with immobilized GST-Ear2 or GST, and specifically bound HisHA-Rasd1 was eluted by heating at 95°C for 10 minutes, and detected by Western blotting with Anti-HA antibody. (B) HisHA-Rasd1 and GST-Ear2 interact in intact mammalian cells. pHisHA-Rasd1 was co-transfected with plasmids expressing either GST-Ear2 or GST into COS-7 cells. GST and GST-Ear2 were captured from the cell lysates by GSH-linked beads and HisHA-Rasd1 bound to the beads were detected as above. (C) COS-7 cells were co-transfected with plasmids expressing HisHA-Rasd1 and GST-Ear2 or GST. Immunoprecipitations were performed with anti-GST antibody. Co-immunoprecipitated HisHA-Rasd1 was detected by Anti-HA antibody. (D and E) Rasd1 and Ear2 form a physiological complex in living cells. Endogenous Rasd1-Ear2 complexes were detected by immunoprecipitating Ear2 from HEK293T cell lysates (D) or mouse brain lysates (E) with goat polyclonal IgG anti-Ear2 antibody, and probing for Rasd1 (D and E, lanes 1). A non-relevant goat polyclonal IgG antibody was used as a negative control (D and E, lanes 2). IP and IB denote immunoprecipitation and immunoblot, respectively.

*Rasd1 and Ear2 interact in vitro and in living cells*- To confirm the specificity of biochemical interaction between Rasd1 and Ear2, we conducted an *in vitro *binding study utilizing transfected COS-7 cells. GST-Ear2 was immobilized on GSH-linked beads. The GST fusion protein containing full-length Ear2 interacted with HA-tagged full-length Rasd1 from the lysate of transfected COS-7 cells (Figure 1A, lane 1), whereas GST itself showed no such interaction (Figure 1A, lane 2).

To test whether Rasd1 and Ear2 interact in intact mammalian cells, we performed co-transfection and co-precipitation experiments. pHisHA-Rasd1 was co-transfected together with pGST-Ear2 into COS-7 cells. As a control, pHisHA-Rasd1 was co-transfected with pxJGST into COS-7 cells. GST-Ear2 or GST from transfected COS-7 cell lysate was immobilized on GSH-linked magnetic particles. Precipitation of GST-Ear2 resulted in the co-precipitation of HisHA-Rasd1 (Figure 1B, lane 1), whereas precipitation of GST-tag alone did not co-precipitate HisHA-Rasd1 (Figure 1B, lane 2). This demonstrates that Rasd1 and Ear2 form a physiological complex in cultured cells. Additionally, a co-immunoprecipitation assay was carried out using lysate derived from COS-7 co-transfected with pHisHA-Rasd1 and pGST-Ear2. As a control, experiments were also conducted using lysate derived from COS-7 cells co-transfected with pHisHA-Rasd1 and pxJGST. Immunoprecipitation of GST-Ear2 with anti-GST antibody resulted in the co-precipitation of HisHA-Rasd1 (Figure 1C, lane 1). There was no non-specific interaction between GST tag and HisHA-Rasd1 (Figure 1C, lane 2).

*Interaction of endogenous Rasd1 and Ear2 was identified in HEK293T cells and the mouse brain*- The existence of endogenous Rasd1-Ear2 complexes was further demonstrated by co-immunoprecipitation of the protein complex from both HEK293T cells and mouse brain. Immunoprecipitation of endogenous Ear2 resulted in the co-immunoprecipitation of Rasd1 (Figure 1D, lane 1 and Figure 1E, lane 1), but immunoprecipitation performed with a non-relevant goat polyclonal IgG antibody did not co-immunoprecipitate Rasd1 (Figure 1D, lane 2 and Figure 1E, lane 2). These experiments confirm the specificity of the endogenous interaction between Rasd1 and Ear2.

**Figure 2:**
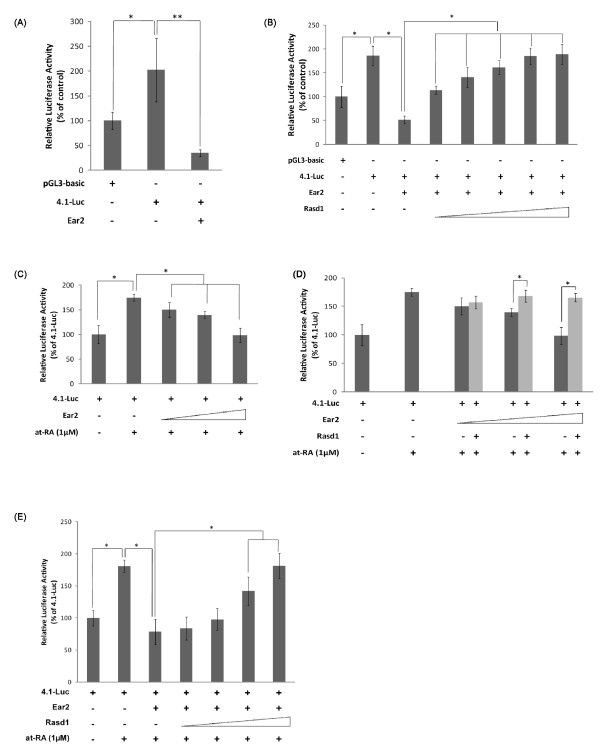
**Rasd1 counteracts repression of renin transcription by Ear2**. (A) Luciferase assay showed that transcription of renin is repressed by Ear2. COS-7 cells were transfected with pGL3-basic (2.0 μg) or p4.1-Luc (2.0 μg) or p4.1-Luc (2.0 μg) and pGST-Ear2 (1.5 μg), and with pSV-β-gal (0.5 μg). p4.1-Luc construct was generated by cloning the 4.1 kb renin promoter into pGL3-basic vector. Relative luciferase activity was normalized against β-gal activity. (B) Rasd1 alleviates Ear2-mediated repression of renin transcription in a dosage dependent manner. COS-7 cells were transfected with a constant amount of p4.1-Luc (2.0 μg), pSV-β-gal (0.5 μg) and pGST-Ear2 (1.5 μg), and with an increasing amount of pHisHA-Rasd1 (0.1, 0.2, 0.5, 1.0 and 1.5 μg). (C) Ear2 attenuates the renin promoter activity induced by retinoic acid in a dosage dependent manner. COS-7 cells were transfected with a constant amount of p4.1-Luc (2.0 μg), pSV-β-gal (0.5 μg) and pHisHA-Rasd1 (1.5 μg), and with an increasing amount of pGST-Ear2 (0.5, 1.0 and 1.5 μg). Renin transcription was induced by all-trans retinoic acid 24 hours post transfection. (D and E) Rasd1 alleviates Ear2-mediated repression of retinoic acid induced renin transcription in a dosage dependent manner. The effect of Rasd1 and Ear2 on retinoic induced renin transcription was tested by measuring the luciferase activity in varying amounts of either Ear2 (D) or Rasd1 (E). The amounts of pGST-Ear2 transfected in *D *were 0.5, 1.0 and 1.5 μg; the amounts of pHisHA-Rasd1 transfected in *E *were 0.2, 0.5, 1.0 and 2.0 μg. * and ** denotes p < 0.05 and p < 0.01 respectively. (A-E) In all luciferase assays, controls were transfected with pGL3-basic (2.0 μg), pSV-β-gal (0.5 μg) and appropriate amounts of the respective carrier vectors.

*Rasd1 alleviates Ear2-mediated repression of renin transcription*- Ear2 binds to the RARE on the renin enhancer and negatively regulates renin gene transcription [13]. The most important regulatory regions of the mouse renin gene reside in 4.1 kb of the renin 5' flanking sequence [6,32]. To explore the possibility that Rasd1 modulates Ear2-mediated transrepression of renin expression, we cloned the 4.1 kb renin promoter and enhancer into the promoterless, enhancerless luciferase pGL3-Basic vector (p4.1-Luc). COS-7 cells were transfected with pGL3-basic or p4.1-Luc or p4.1-Luc and pGST-Ear2. We first validated that Ear2 acts as a negative regulator on renin gene transcription [13] in COS-7 cells (Figure 2A). We then proceeded to test if Rasd1 was able to modulate renin transcription in the presence of Ear2. Our results show that Rasd1 alleviates the Ear2-mediated transcriptional repression of renin promoter activity in a dosage-dependent manner (Figure 2B). It has also been reported that Ear2 is able to downregulate retinoic acid-induced renin transcription [13]. We validated this result (Figure 2C) and tested if Rasd1 was able to alleviate Ear2-mediated transcriptional repression of retinoic acid-induced renin expression. Our results suggest that Rasd1 is able to alleviate Ear2-mediated transcriptional repression of retinoic acid-induced renin promoter activity in a dosage dependent manner (Figure 2D and 2E).

**Figure 3:**
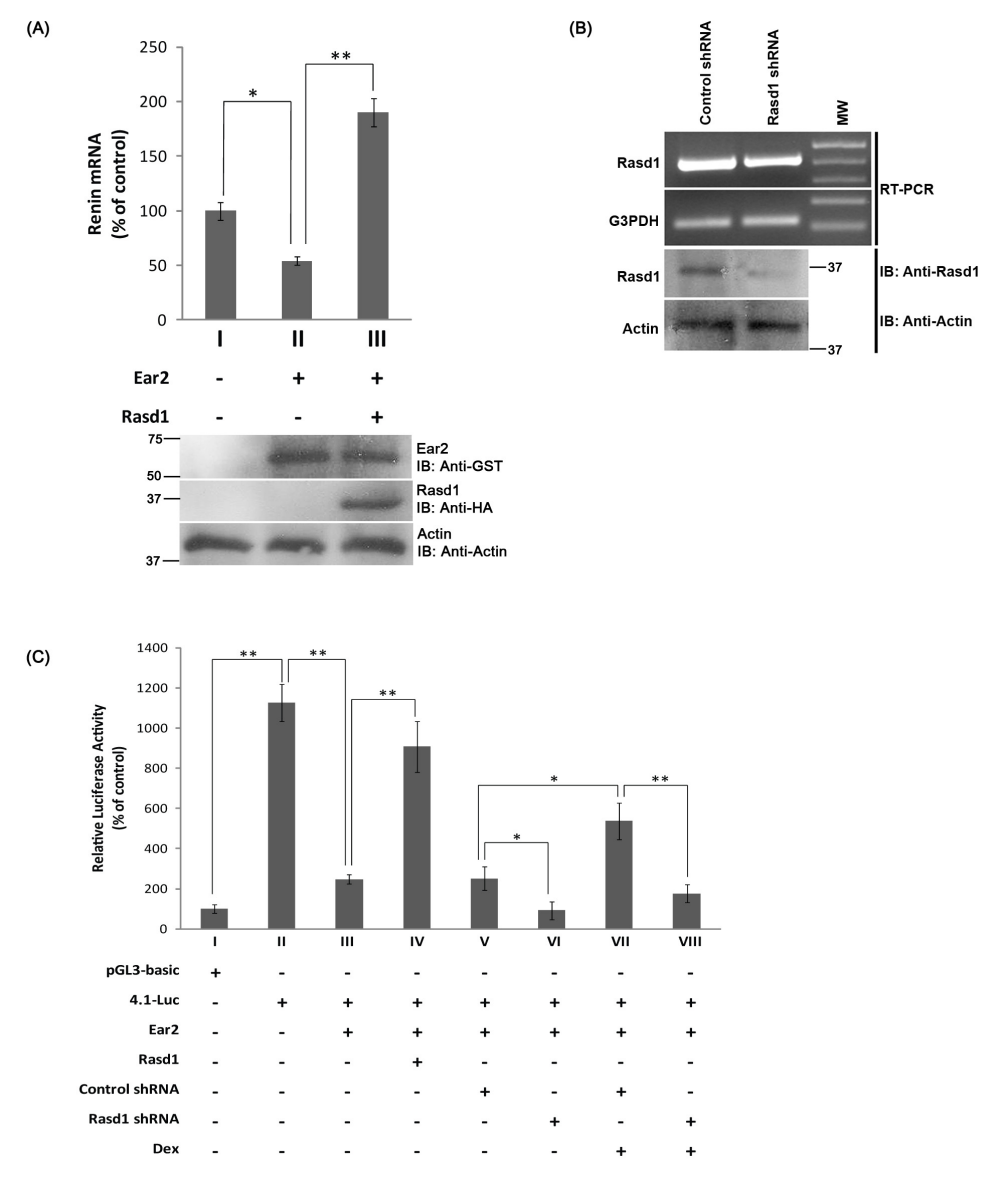
**Transcriptional repression of renin by Ear2 can be modulated by alternating the endogenous level of Rasd1**. (A) Real-time RT-PCR was conducted to determine level of endogenous renin transcription when Rasd1 was overexpressed. As4.1 cells were transiently transfected with pxJGST (2.0 μg) and pcDNA4.0 (3.5 μg) (bar I), or pGST-Ear2 (2.0 μg) and pcDNA4.0 (3.5 μg) (bar II), or pHisHA-Rasd1 (3.5 μg) and pcDNA4.0 (3.5 μg) (bar III). Representative immunoblots demonstrated Ear2 and Rasd1 transgene expression 48 hours after transfection (lower panels). (B) Semi-quantitative RT-PCR showed Rasd1 knockdown in the As4.1 cells. As4.1 cells were transfected with Rasd1 shRNA or control shRNA (4.0 μg). Cells were selected with puromycin 24 hours post transfection and harvested 48 hours post transfection. The cDNA amplified were run on a 1.5% agarose gel with G3PDH as an internal control (upper panels). A representative immunoblot demonstrated endogenous Rasd1 protein knockdown by Rasd1 shRNA (lower panel). Proteins were detected with anti-Rasd1 and anti-actin antibodies. Quantification of the immunoblots by densitometer revealed that Rasd1 protein levels, normalized against actin protein levels, were knocked down by more than 48% (lower panels). (C) Luciferase study showed that dexamethasone treatment counteracts the effect of Rasd1 shRNA knockdown. As4.1 cells were transfected with p4.1-Luc (2.0 μg), pGST-Ear2 (1.5 μg), pHisHA-Rasd1 (1.5 μg), Rasd1 shRNA or control shRNA as indicated (4.0 μg), together with pSV-β-gal (0.5 μg). Controls were transfected with pGL3-basic (2.0 μg), pSV-β-gal (0.5 μg) and appropriate amounts of the respective carrier vectors. Cell were selected with puromycin (2 μg/ml) and induced by dexamethasone (100 nM) 24 hours after transfection. Cells were harvested and analyzed for luciferase activity 48 hours after transfection. Relative luciferase activity was normalized against β-gal activity. * and ** denotes p < 0.05 and p < 0.01, respectively.

*Rasd1 modulates endogenous renin gene expression by interacting with Ear2*- We also investigated the effects of Ear2 and Rasd1 on endogenous renin gene expression in As4.1 cells. As4.1 cells express endogenous renin, as well as Rasd1 and Ear2. Real-time RT-PCR showed that renin mRNA levels were lower in the cells over-expressing Ear2. Renin mRNA in As4.1 cells over-expressing Ear2 were reduced to about 48% of the control (Figure 3A, compare bars I and II). When pHisHA-Rasd1 was co-transfected with pGST-Ear2 into As4.1 cells, the repression of renin transcription by Ear2 was inhibited (Figure 3A, compare bars II and III). Renin mRNA levels increased to almost twice the amount of control when Rasd1 was over-expressed (Figure 3A, compare bars I and III). This spike in renin gene expression when Rasd1 was over-expressed could be attributed to the presence of abundant Rasd1, which might have counteracted the repression of renin transcription by both endogenous and transfected Ear2.

We proceeded to test if alteration of Rasd1 levels in As4.1 cells is capable of modulating Ear2-mediated repression of renin transcription. As4.1 cells are known to express high level of renin [33]. We first demonstrated that Rasd1 shRNA, but not control shRNA, effectively knocked down endogenous expression of Rasd1 mRNA (Figure 3B, upper panel) and protein (Figure 3B, lower panel) in As4.1 cells. Quantifications with the densitometer indicated that protein levels were knocked down by more than 48%. In addition, we demonstrated that shRNA-mediated knockdown of Rasd1 results in a further repression of Ear2-mediated renin transcription (Figure 3C, compare bars V and VI). Because dexamethasone is known to induce Rasd1 expression [28], we examined whether dexamethasone could counteract the effects of shRNA-mediated Rasd1 knockdown. In the presence of dexamethasone, Ear2-repressed renin transcription was alleviated (Figure 3C, compare bars V and VII). This resembled the results we observed from Rasd1 over-expression (Figure 3C, compare bars III and IV). Treatment with dexamethasone counteracted the effects of Rasd1 shRNA on the Ear2-mediated repression of renin transcription (Figure 3C, compare bars VI and VIII); however, renin transcription levels were restored to lower levels than that of treatment with dexamethasone alone (Figure 3C, bar VII). Our experiments suggest that transcriptional repression of renin by Ear2 can be modulated by alternating the level of Rasd1 in As4.1 cells.

**Figure 4:**
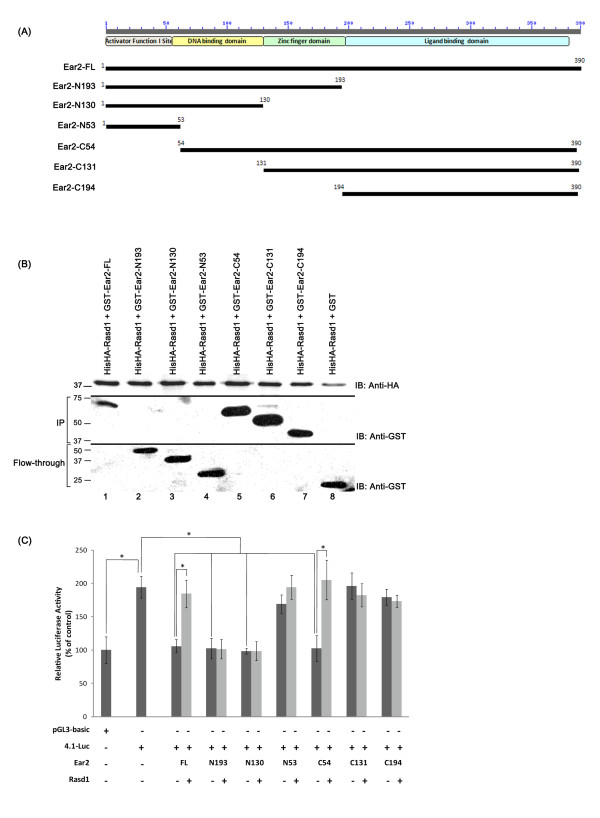
**Ear2 ligand binding domain interacts with Rasd1**. (A) Schematic diagram showing the truncated constructs of Ear2. Ear2 contains 4 main domains: an activator function I site, a DNA binding domain, a zinc finger domain and a ligand binding domain. Full length (FL) Ear2 has 390 amino acids and each of the six truncated Ear2 constructs is represented by their amino acid numbers on the left. (B) Immunoblot analysis showed that Rasd1 binds to Ear2 ligand binding domain. COS-7 cells were co-transfected with pHisHA-Rasd1 and the indicated pGST-Ear2 constructs. HisHA-Rasd1 from the cell lysates was immobilized on Ni-NTA beads and GST-Ear2 constructs bound to the complexes were eluted by heating at 95°C for 10 minutes, and detected by immunoblotting with anti-GST (lanes 1 and 5-7). GST-Ear2 constructs not co-precipitated with HisHA-Rasd1 were detected in the flow-through (lanes 2-4 and 8). (C) Luciferase study showed that Ear2 ligand binding domain and DNA binding domain are required for interacting with Rasd1 to modulate renin transcription. COS-7 cells were transfected with p4.1-Luc (2.0 μg), pGST-Ear2 (1.5 μg) or plasmids expressing Ear2 truncated constructs and pHisHA-Rasd1 (1.5 μg) as indicated, together with pSV-β-gal (0.5 μg). Total DNA transfected was held constant with the respective carrier vector plasmids. Controls are transfected with pGL3-basic (2.0 μg), pSV-β-gal (0.5 μg) and appropriate amounts of the respective carrier vectors. Relative luciferase activity was normalized against β-gal activity. Ear2 constructs that were missing their DNA binding domains (Ear2-N53, Ear2-C131, Ear2-C194) did not significantly repress renin transcription. Rasd1 alleviated Ear2-repressed renin transcription only if Ear2 ligand binding domain is present (Ear2-FL, Ear2-C54). * denotes p < 0.05.

*Ear2 interacts with Rasd1 via its ligand binding domain*- Ear2 is a 390 amino acid nuclear hormone receptor and is known to contain several domains, including an activator function I site (residues 1-53), a DNA-binding domain (residues 54-130), a linker region (residues 131-193) and a ligand binding domain (residues 194-376). To elucidate how Rasd1 interacts with Ear2, we generated six GST-Ear2 truncated constructs (Figure 4A) and used them to carry out co-precipitation assays against full-length HisHA-Rasd1. We found that only full-length Ear2 (Figure 4A, Ear2-FL) and Ear2 truncated constructs that contain the ligand binding domain (Figure 4A, Ear2-C54, Ear2-C131 and Ear2-C194) co-precipitated with HisHA-Rasd1 (Figure 4B, lane 1 and lanes 5-7). This indicates that the C-terminus ligand binding domain, containing amino acids 194-390, is required for the binding of Ear2 to Rasd1.

*Ear2 ligand binding domain is required for Rasd1 to alleviate Ear2-mediated repression of renin transcription*- To further investigate the binding of Ear2 on the renin enhancer [13], we performed luciferase assay using the six truncated constructs of Ear2. Ear2 deletion constructs devoid of the DNA binding domain markedly lost their ability to repress renin transcription (Figure 4C, bars with Ear2-N53, Ear2-C131 and Ear2-C194). This result is in accordance with previous report which shows that Ear2 with a mutated DNA binding domain is unable to bind to the RARE and ineffective in inhibiting the activity of the renin promoter [13].

When Rasd1 was added, we observed that Rasd1 alleviated Ear2-mediated transcriptional repression of renin promoter activity only in Ear2 constructs with intact DNA and ligand binding domains (Figure 4C, bars with Ear2-FL and Ear2-C54). This further confirms our previous observations that Rasd1 interacts with the ligand binding domain of Ear2. It also shows that the specific binding of Rasd1 to Ear2 is essential for Rasd1 to counteract Ear2-mediated transrepression of renin promoter. In addition, it was observed that the activator function I domain (Figure 4A, Ear2-N53) of Ear2 was not required for its repression of renin promoter activity. Deletion of the activator function I domain has no influence on either the Ear2-mediated repression or the Rasd1-mediated alleviation of renin promoter activity (Figure 4C, lanes with Ear2-FL and Ear2-C54).

**Figure 5:**
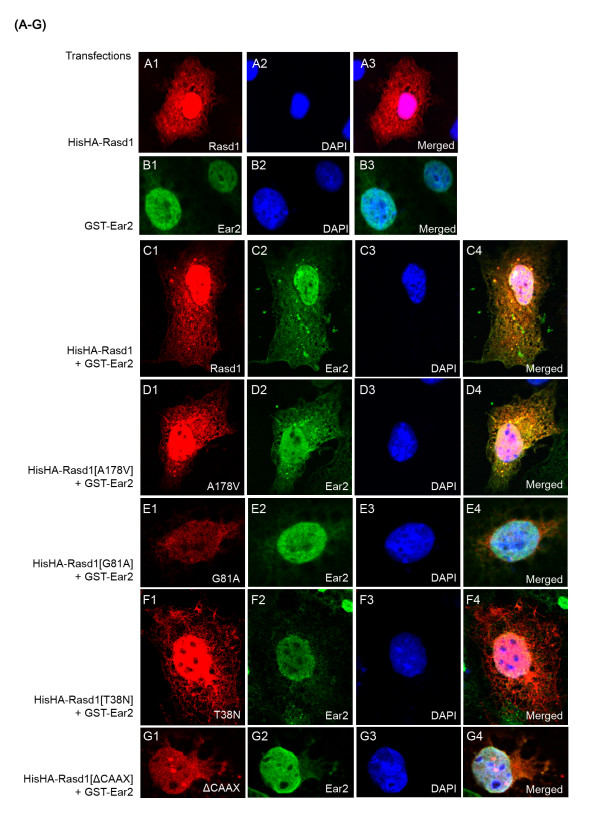
**Effects of wild-type and Rasd1 mutants on nuclear-cytoplasmic distribution of Ear2**. (A-G) Immunofluorescence staining of COS-7 cells transfected with pHisHA-Rasd1 (A1-3) or pGST-Ear2 (B1-3) or pHisHA-Rasd1 (wild type or mutants) + pGST-Ear2 (C1 to G4). HisHARasd1 (wild type or mutants) were detected with anti-HA antibody and visualized with AlexaFluor 568 (red); GST-Ear2 was labeled with anti-GST AlexaFluor 488 (green); nucleus was labeled with 4',6-diamidino-2-phenylindole (DAPI) (blue). Confocal imaging showed that HisHA-Rasd1 is present in both the nucleus and cytoplasm (A1-3). GST-Ear2 is located mainly in the nucleus (B1-3), but when co-transfected together with HisHA-Rasd1, GST-Ear2 is detected in both the nucleus and cytoplasm (C2).

*Rasd1 and Ear2 colocalize in the nuclei *- Rasd1 and Ear2 must both be physically present in the same cellular compartment to form a physiological complex. We therefore decided to examine the localization of Rasd1 and Ear2 in transfected COS-7 cells. When transfected alone, HisHA-Rasd1 was expressed in both the cytoplasm and the nucleus (Figure 5, A1-3), while GST-Ear2 was present mainly in the nucleus (Figure 5, B1-3). When GST-Ear2 and HisHA-Rasd1 were co-expressed, it was observed that HisHA-Rasd1 and GST-Ear2 colocalized in both the cytoplasm and the nucleus (Figure 5, C1-4). It is interesting to note that the amount of GST-Ear2 present in the cytoplasm was dramatically increased in the presence of HisHA-Rasd1 (compare Figure 5, B1 with C2); there was no noticeable change in the distribution of HisHA-Rasd1 in the presence or absence of GST-Ear2 (compare Figure 5, A1 with C1).

*Missense mutations in Rasd1 abolished its physical and functional interaction with Ear2*- Rasd1 is a brain-enriched G protein that belongs to the RAS superfamily [34]. RAS superfamily members consists of GTPases with high conservation in sequence and structural organization, especially within their GTP binding pockets -G1, G3, G4 and G5 boxes [34]. Most RAS subfamily proteins also possess a C-terminus CAAX box, which undergoes post-translational isoprenylation and regulates the subcellular localization and function of the proteins [35]. To investigate whether GTPase activity, GDP-GTP exchange by GEF and post-translational isoprenylation of Rasd1 are required for its alleviation of Ear2-mediated repression of renin transcription, we generated four Rasd1 mutants- Rasd1[A178V], Rasd1[G81A], Rasd1[T38N] and Rasd1[ΔCAAX]. Rasd1[A178V] [36] contains a single nucleotide mutation in the G5 domain, disrupting its guanyl nucleotide binding pocket. Rasd1 [A178V] behaves functionally as a constitutively active signal transducer. Rasd1[G81A] possesses a point mutation that lies on a highly conserved glycine residue in the G3 box [37]. In human HRAS, the corresponding G60A mutation drastically reduces its GTPase activity [38]. Rasd1[T38N] contains a mutation in the G1 box, corresponding to human RAS[S17N]. Human HRAS[S17N] binds guanine nucleotide exchange factors (GEFs) but does not catalyze the release of GDP, thus effectively blocking the activation of RAS [39,40]. To explore the potential role of isoprenylation in the functional properties of Rasd1, we generated Rasd1[ΔCAAX]. This mutant contains a premature termination codon that gives rise to a truncated Rasd1 devoid of the CAAX box.

The Rasd1 mutant constructs were co-transfected with Ear2 and their cellular localization was examined. Rasd1[A178V] and Rasd1[T38N] exhibited a similar distribution to that of wild type Rasd1 and were present in both the nucleus and cytoplasm (Figure 5, A1, D1 and F1). Rasd1[G81A] and Rasd1[ΔCAAX] were located only in the nucleus (Figure 5, E1 and G1). When co-expressed with Ear2, the amount of Ear2 present in the cytoplasm was visibly increased in the presence of Rasd1[A178V] (compare Figure 5, B1 and D2). On the other hand, Rasd1[G81A], Rasd1[T38N] and Rasd1[ΔCAAX] did not alter the distribution of Ear2. Ear2 was still mostly located in the nucleus in the presence of these Rasd1 mutants (Figure 5, E2, F2, and G2).

**Figure 6:**
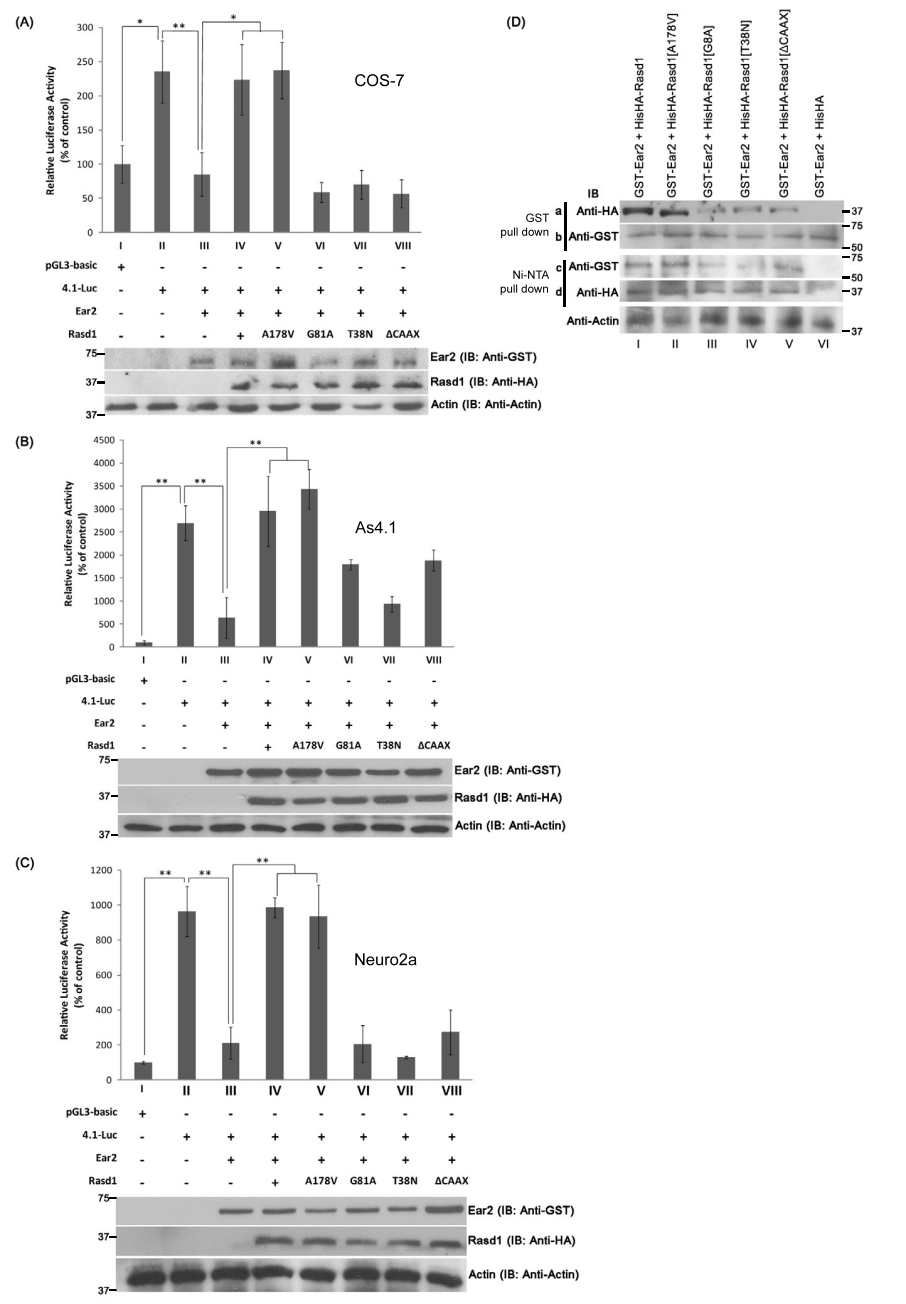
**(A-C) Effects of wild-type and Rasd1 mutants on Ear2-mediated renin transcription**. Luciferase studies showed that: A, COS-7; B, As4.1; C, Neuro2a. Rasd1[A178V] mutant alleviated Ear2-mediated repression of renin transcription in a magnitude similar to wild-type Rasd1 (compare bars IV and V), whereas Rasd1[G81A], Rasd1[T38N] and Rasd1[ΔCAAX] did not significantly alleviate Ear2-mediated repression of renin transcription (bars VI-VIII). (A, B and C, lower panels) Western blots showed similar expression efficiency of proteins. In all blots, expression of actin is shown as a protein loading control. * and ** denotes p < 0.05 and p < 0.01, respectively. (D) Co-precipitation followed by immunoblot showed that Rasd1[G81A], Rasd1[T38N] and Rasd1[ΔCAAX] mutations interfere with their ability to interact with Ear2. COS-7 cells were co-transfected with pGST-Ear2 and the respective Rasd1 construct expressing plasmids or the empty vector. Precipitation of GST-Ear2 with GSH-linked magnetic beads co-precipitated with Rasd1 proteins expressed from each construct (panel a, lanes I-V). However, the amount of HAHis-Rasd1[G81A], HAHis-Rasd1[T38N] and HAHisRasd1[ΔCAAX] co-precipitated were significantly less than that of HAHis-Rasd1 and HAHis-Rasd1[A178V] (compare panel a, lanes I and II with lanes III-V). Similarly, co-precipitation assays using magnetic Ni-NTA beads revealed that the amount of GST-Ear2 co-precipitated with HAHis-Rasd1[G81A], HAHis-Rasd1[T38N] and HAHisRasd1[ΔCAAX] were significantly less than that of HAHis-Rasd1 and HAHis-Rasd1[A178V] (compare panel c, lanes I and II with lanes III-V).

We proceeded to perform luciferase assays to investigate the effect of the expression of wild-type versus various mutant Rasd1 proteins on Ear2-mediated renin promoter activity in three cell lines, including COS-7 (monkey kidney fibroblast cell line), As 4.1 (mouse juxtaglomerular cell line) and Neuro2a (mouse neuroblastoma cell line). Both As4.1 and Neuro2a cells are known to endogenously express renin [33,41], whereas no endogenous expression of renin is observed in COS-7cells [12]. In agreement with this, the 4.1-Luc reporter construct exhibited an eleven fold and four fold higher luciferase reporter activity in As4.1 and Neuro2a cells, respectively, than in COS-7 cells (compare Figure 6 bars: BII and AII; CII and AII). Luciferase reporter assay showed that over-expression of Ear2 suppressed renin promoter activity in all three cell lines (compare Figure 6 bars: AII and AIII; BII and BIII; CII and CIII) and over-expression of Rasd1 alleviated Ear2-mediated down regulation of the renin promoter (compare Figure 6 bars: AIII and AIV; BIII and BIV; CIII and CIV). Likewise, Rasd1[A178V], a constitutively active mutant, alleviated Ear2-mediated down-regulation of the renin promoter in a magnitude similar to that of wild-type Rasd1 (Figure 6A, compare bars: AIII and AIV; BIII and BIV; CIII and CIV). In contrast, Rasd1[G81A], Rasd1[T38N] and Rasd1[ΔCAAX] did not significantly alleviate Ear2-mediated down-regulation of renin transcription (Figure 6A, compare bars III, IV and VI-VIII), thus indicating that the GTP hydrolysis activity of Rasd1, GDP-GTP exchange by GEF and isoprenylation of Rasd1 are required for counteracting Ear2-mediated repression of renin transcription. To investigate whether Rasd1[A178V], Rasd1[G81A], Rasd1[T38N] and Rasd1[ΔCAAX] mutations will interfere with their ability to interact with Ear2, we conducted co-immunoprecipitation analyses. The immunoblotting results showed that significantly less Rasd1[G81A], Rasd1[T38N] and Rasd1[ΔCAAX] were co-precipitated with immobilized GST-Ear2 when compared to wild type Rasd1 and Rasd1[A178V] (compare Figure 6D, panel a, lanes I and II with lanes III-V). Similarly, noticeably less GST-Ear2 co-precipitated with immobilized Rasd1[G81A], Rasd1[T38N] and Rasd1[ΔCAAX] compared to immobilized wild type Rasd1 and Rasd1[A178V] (compare Figure 6D, panel c, lanes I and II with lanes III-V). These results suggest that mutation of amino acid 81, 38 and deletion of the CAAX box of Rasd1 affects the ability of Rasd1to interact with Ear2.

## Discussion

In our present study, we identified Ear2 as a direct interacting target of Rasd1. The interaction between Ear2 and Rasd1 was confirmed *in vitro *and in living cells. Ear2 interacts with Rasd1 via its ligand binding domain. In addition, we demonstrated that Rasd1 is able to alleviate both retinoic acid-dependent and independent Ear2-mediated transcriptional repression of renin promoter activity. Furthermore, we showed that the Rasd1 mutants, Rasd1[G81A], Rasd1[T38N] and Rasd1[ΔCAAX], which have reduced physical interaction with Ear2, are ineffective in counteracting repression of renin transcription by Ear2. Our current data suggests that Rasd1 may facilitate the translocation of Ear2 from the nucleus to the cytoplasm. Alternatively, Rasd1 may bind to Ear2 and render it inactive in the transrepression of renin promoter. Further experiments are required to delineate the detailed mechanism by which Rasd1 counteracts repression of renin transcription by Ear2.

Ear2 is involved in the negative regulation of renin gene transcription [13]. Renin is the rate-limiting enzyme in the renin-angiotensin enzymatic cascade which leads to the production of the bioactive product Angiotensin-II [2,3]. However, despite the efforts aimed at characterizing the renin enhancer, its functional relevance *in vivo *has yet to be determined. In our study, we showed that Rasd1 interacts with Ear2, and this finding prompted us further to investigate the role of Rasd1 on Ear2-mediated renin transcription. Interestingly, we found that Rasd1 alleviated Ear2-repressed renin transcription in a dosage-dependent manner and that this novel function of Rasd1 required a specific interaction between Rasd1 and the ligand binding domain of Ear2.

The renin transcriptional enhancer contains both positive and negative regulatory elements [9-12]. Among them is an unusual RARE with TGACC tandem repeats separated by 10 nucleotides (TGACCT-DR10-TGACCT), which is required for both retinoic acid-mediated and retinoic acid-independent activity of the enhancer [9]. Ear2 has been shown to mediate negative regulation of renin transcription by competing with RAR/RXR for its binding to RARE [9,13]. Our experiments showed that Rasd1 is able to relieve Ear2-mediated transcriptional repression of renin via direct interaction with Ear2 both in the presence or absence of retinoic acid. It is possible that Rasd1 acts by binding directly to Ear2, sequestering Ear2 from the nucleus to the cytoplasm, and removing the inhibitory effect of Ear2 by allowing other stimulatory factors to bind to the renin enhancer. Rasd1 contains a bipartite nuclear localization [36], and has been shown to be present in both nucleus and cytosol [36]. Our immunofluorescence and confocal studies showed that a significant amount of Rasd1 is localized in the nuclei. Interestingly, when Rasd1 was co-transfected with Ear2, it resulted in the movement of a significant amount of Ear2 from the nucleus into the cytosol. This suggests that Rasd1 may play a role in regulating Ear2 nucleus-cytosol distribution.

Rasd1 belongs to the RAS superfamily. Members of the RAS superfamily are highly conserved in sequence and show high degrees of homology across species. Similar to other members of the RAS superfamily, Rasd1 possesses five highly conserved motifs for GTP binding and hydrolysis (G1-G5), an effector loop that mediates protein-protein interactions, and a C-terminus CAAX box that serves as a consensus site for the isoprenylation required for membrane localization [34]. Despite extensive similarities to members of the Ras family, Rasd1 also possesses several differences, including an extended carboxyl terminus variable cationic domain and a high basic net isoelectric point [42]. These differences indicate that Rasd1 may play diverse biological roles that have yet to be discovered. This study demonstrates that Rasd1 serves a novel role in the regulation of Ear2-mediated renin transcription. In view of the highly conserved sequence and function of members of the RAS superfamily, we generated several Rasd1 mutants in an attempt to elucidate the molecular basis of the alleviation of Ear2-mediated repression of renin transcription by Rasd1. As expected, Rasd1[A178V], a constitutively active mutant of Rasd1, was able to alleviate Ear2-mediated transcriptional repression of renin promoter activity. In contrast, Rasd1[G81A], Rasd1[T38N] and Rasd1[ΔCAAX] did not alleviate Ear2-mediated repression of renin transcription. This suggests that GTPase activity, GDP-GTP exchange by GEFs, isoprenylation and possibly, the targeting of Rasd1 to the cell membrane are all involved in this function. Mutant H-RAS[S17N] inhibits Ras dependent pathways and has been shown to have a much higher affinity for GDP than GTP [40]. As expected, the corresponding Rasd1[T38N] mutant was inactive with respect to the upregulation of renin transcription. Similarly, the CAAX box-deficient mutant, Rasd1[ΔCAAX], was unable to upregulate renin transcription, thus suggesting that isoprenylation and membrane colocalization of Rasd1 is necessary. Likewise, mutant Rasd1[G81A], which binds with reduced affinity to GAPs and possesses attenuated GTPase activity [38], did not alleviate Ear2-mediated repression of renin transcription. We propose a few plausible explanations for this observation. The binding of Rasd1 to Ear2 and the subsequent translocation and retention of Ear2 in the cytosol by Rasd1 may require the GTP hydrolysis activity of Rasd1. Thus, Rasd1[G81A], with reduced GTPase activity, does not effectively bind to Ear2 and translocate it out of the nucleus to remove the repression of Ear2 on the renin promoter. Alternatively, the lack of functional activity of Rasd1[G81A] may be due to a reduced affinity of Rasd1[G81A] for Ear2. In H-RAS, the G60A mutation perturbs GTP-induced conformational change and abolishes biological activity [43]. In another example, a corresponding mutant of EF-Tu, EF-Tu[G83A], has increased GTPase activity and a reduced binding affinity for aa-tRNA [44].

When we conducted interaction studies, it was discovered that Rasd1[G81A], Rasd1[T38N], Rasd1[ΔCAAX] had visibly weaker interactions with Ear2 compared to wild type Rasd1 and Rasd1[A178V]. Immunofluorescence and confocal studies further show that Rasd1[G81A], Rasd1[T38N] and Rasd1[ΔCAAX] were ineffective in translocating and retaining Ear2 in the cytoplasm. It is also interesting to note that while wild type Rasd1, Rasd1[A178V] and Rasd1[T38N] were present in both the nucleus and cytoplasm, Rasd1[G81A] and Rasd1[ΔCAAX] were localized mainly in the nucleus, suggesting that GTPase activity and isoprenylation of Rasd1 affects Rasd1 nuclear-cytoplasmic distribution. Taken together, our data implies that GTPase activity, GDP-GTP exchange and isoprenylation and membrane localization of Rasd1 is required for its functional activity in the mediation of Ear2-repressed renin transcription. Rasd1 mutants that possess these defects were rendered ineffective in the alleviation of Ear2-repressed renin transcription, possibly due to a weakened interaction with Ear2 and/or a reduced effectiveness in the translocation of Ear2 from the nucleus to the cytoplasm.

Many physiological functions, such as blood pressure, exhibit endogenous rhythmic variability corresponding to a day-night cycle of close to 24 hours and driven by the circadian system, which consists of central oscillators in the SCN and peripheral oscillators in organs like the heart, pancreas, kidneys and liver [45-47]. The expression of *Rasd1 *in the SCN exhibits a circadian rhythm [29]. This leads us to speculate about the relevance of *Rasd1*'s involvement in the regulation of Ear2-mediated renin transcription. We postulate that *Rasd1 *might be a player in the regulation of the daily circadian pattern of blood pressure through its interaction with Ear2 and consequent effect on renin transcription. Interestingly, Ear2^-/- ^mice displayed reduced efficiency to photic and feeding time cues entrainment [25]. Ear2 is also required for the normal rhythmic expression of *Per1 *in the forebrain [25,48]. Might Rasd1 and Ear2, together with other clock genes, work in a currently unknown, complex interacting cascade to affect the circadian control of the renin-angiotensin pathway?

There is substantial evidence supporting the existence of a complete and functionally competent renin-angiotensin system in the brain [3,49-51]. In addition to the classical roles of peripheral angiotensins, various studies have shown that central angiotensins are also involved in less conventional functions, such as sexual behavior [52,53], stress [54-56], and learning and memory [52]. There are also studies that show that the renin-angiotensin system is involved in neurological disorders, including Alzheimer's [57-60] and Parkinson's [60,61] disease. Our identification of this novel regulatory mechanism of the renin-angiotensin system opens exciting possibilities for future research and will be of considerable interest for clinical application. The renin-angiotensin system, classically known for its involvement in the regulation of blood pressure, has been a target in the development of drugs for the treatment of hypertension. Hypertension is in turn a potent risk factor for cardiovascular diseases, such as stroke, atherosclerosis, and heart attacks [62-64]. Clinically, inhibitors of the renin-angiotensin system are observed to be effective antihypertensive treatments [65-67]. Targeting the brain renin-angiotensin system may also be a viable option for the treatment of mood disorders, cognitive dysfunctions and even neurodegenerative diseases. Obtaining a better understanding of the regulation of the renin-angiotensin system may eventually lead to the production of more effective drugs.

## Conclusions

We conclude that Rasd1 and Ear2 interact physically and functionally. This interaction counteracts the repression of renin transcription by Ear2. Since Ear2 is also known to play an important role in brain development and circadian control, it remains to be determined how the interplay between these two proteins regulates spatial-temporal developmental and physiological processes. The results of this study could provide mechanistic insights into Rasd1's ability to modulate these processes through its interaction with Ear2.

## Methods

*Yeast two-hybrid screening*- All vectors, yeast strains, reagents and methods were derived from the MATCHMAKER Two-Hybrid System 3 (Clontech, Palo Alto, CA, USA). PCR-generated 843bp full length mouse *Rasd1 *cDNA was inserted into *Nde*I and *Eco*RI digested vector pGBKT7 as an in-frame fusion with the DNA binding domain of GAL4 at its 3' end, and the resulting plasmid was designated pGBKT7-Rasd1. The correct insertion of *Rasd1 *was confirmed by restriction analysis and verified by sequencing. pGBKT7 carries the yeast nutritional selection marker *TRP1*. Library screening was performed using Rasd1-Gal4 DNA binding domain fusion protein expressed from pGBKT7-Rasd1 as the bait. Absence of autonomous activation of reported genes in yeast was verified by bait before library screening. Mouse brain MATCHMAKER cDNA library culture (Clontech, Palo Alto, CA, USA) was used as prey. The library was cloned into pACT2 vector that encodes for the GAL4 transactivation domain and *LEU2 *as the yeast nutritional selection marker. Yeast strains used were AH109 and Y187. AH109 contains two nutritional reported genes for adenine and histidine. Yeast transformations were performed using high-efficiency lithium acetate/poly-ethylene glycol method [68]. Yeast AH109 was transformed with pGBKT7-Rasd1 and nutritionally selected on synthetic dropout (SD)-trp plates. Mouse brain cDNA library cloned in pACT2 was then co-transformed into yeast AH109 that contained pGBKT7-Rasd1 and plated on SD-trp-leu medium to nutritionally select for the presence of both proteins. Putatively positive colonies were selected on SD-ade-his-trp-leu plates and assayed for β-galactosidase activity by use of 5-bromo-4-chloro-3-indolyl-β-D-galactopyranoside (X-gal) as a substrate. Secondary screens were performed in a similar manner to minimize false positives. Positive colonies were subcultured and grown to saturation in SD-leu medium to expel the bait plasmid. Library plasmids from positive interacting colonies were recovered and sorted into groups according to PCR product sizes with primers MATCHMAKER 5' AD LD-Insert screening amplimer and 3' AD LD-Insert screening amplimer and restriction digestion pattern with *Hpa*II. The clones were then sequenced using GAL4 activation domain sequencing primer. BLAST searches (National Centre for Biotechnology Information, NCBI) were performed to identify these clones. The specificity of protein-protein interaction was further tested by yeast mating according to manufacturer's protocol. Y187 was transformed with either pGBKT7-Rasd1 or pGBKT7 vector, and nutritionally selected in medium lacking tryptophan. AH109 was transformed with either pACT2-Ear2 or pACT2 vector alone and grown in medium lacking leucine. To verify the putative positive clones, the transformed yeast strain AH109(MATa) were then mated with Y187(MATα) and grown in medium lacking tryptophan and leucine. The cells were then replica plated on SD-ade-his-trp-leu and re-assayed for β-galactosidase activity.

*Plasmids*- The pHisHA-Rasd1 construct was generated by cloning the 843 bp coding region of *Rasd1 *into *Kpn*I and *Eco*RI digested pcDNA4/HisMax^©^B (Stratagene) vector. Rasd1 was tagged in frame with the 5' polyHis tag and Xpress epitope. In addition, a hemagglutinin (HA) tag was added in frame with the coding region of *Rasd1 *at its 3' terminal using polymerase chain reaction (PCR). Rasd1[ΔCAAX] was also cloned into *Kpn*I and *Eco*RI digested pcDNA4/HisMax^©^B. Site-directed mutagenesis based on overlap extension PCR [69] using two common flanking primers along with individual overlapping mutagenic primers was used to generate the Rasd1 mutant constructs A178V, G81A and T38N. The resulting PCR products were cloned into *Kpn*I and *Eco*RI digested pcDNA4/HisMax^©^B. pGST-Ear2 construct was generated by cloning the 1173bp *Ear2 *coding region into pxJGST vector, tagged in frame with a 5' glutathione-S-transferase (GST) tag with the inFusion cloning kit (Clontech), using *Not*I restriction site. GST-Ear2 truncated constructs carrying DNA sequences corresponding to amino acids 1-193, 1-130, 1-53, 54-390, 131-390 and 194-390 of Ear2 were also cloned into *Not*I digested pxJGST vector. pxJGST vector was derived from pxJ-FLAG-S vector [70]. p4.1-Luc construct was generated by cloning the 4.1kb renin promoter into *Nhe*I and *Hind*III digested pGL3-basic vector (Promega). Rasd1 shRNA knockdown plasmid construct was generated by annealing oligonucleotides targeted at the open reading frame of Rasd1 and inserting the annealed oligonucleotides into *Bgl*II and *Hind*III digested pSUPER.puro (Oligoengine) vector. Control shRNA was generated by inserting randomly jumbled sequences of the Rasd1 shRNA oligonucleotide sequences into *Bgl*II and *Hind*III digested pSUPER.puro. All clones were checked by restriction analysis and verified by sequencing.

*Cell culture and Transfection*- COS-7 cells (American Type Culture Collection, ATCC CRL-1651) were maintained in RPMI1640 (HyClone). As4.1 cells (ATCC CRL-2193) were maintained in DMEM (ATCC). Neuro2a cells were maintained in MEM (Hyclone). HEK293T cells were maintained in DMEM (Hyclone). All media was supplemented with 10% FBS, penicillin (100 U/mL) and streptomycin (100 mg/mL). Cells (3.0-3.5 × 10^5^) were cultured in each well of 6-well culture plates for 24 hours before transfection. For pull-down assays, confocal studies and experiments that measure endogenous renin levels, cells were transfected with 2.5 μg of pGST-Ear2 per well. In co-transfection experiments, pHisHA-Rasd1 (3.5 μg) was transfected together with pGST-Ear2 (2.0 μg). For luciferase assays, cells were transfected with p4.1-Luc or pGL3-basic (2.0 μg), pGST-Ear2 (0.5- 1.5 μg), pHisHA-Rasd1 (0.1- 1.5 μg), pSV-β-gal (0.5 μg) in the combinations as indicated in the figure legends. Total amount of transfected DNA in all transfections were held constant with the appropriate amounts of respective empty vector added to the plasmid mix. shRNA knockdown experiments were performed by transfecting Rasd1 shRNA or non-targeting control shRNA (2 μg/ml). Selection with puromycin (2 μg/ml) and dexamethasone (100 nM) treatment was carried out 24 hours post transfection, and cells were harvested 48 hours post transfection. Knockdown efficiency was determined by semi-quantitative RT-PCR and Western blotting. Quantification of immunoblots was measured using the GS-800 calibrated densitometer (Bio-Rad). Transfection was carried out with DNA (μg) to Lipofectamine 2000 (μl) (Invitrogen) ratio of 1:2, according to manufacturer's instructions.

*Protein binding assays*- In GST pulldown assays, COS-7 cells were transfected separately with pGST*-*Ear2 and pHisHA*-*Rasd1. Cells were harvested 40 to 48 hours after transfection and lysed with lysis buffer (1% Triton-X 100, 15% Glycerol, 1 mM phenylmethylsulphonyl fluoride, 150 mM NaCl, 100 mM Tris, pH 7.4, protease inhibitor (Roche)) for 20 minutes to 1 hour, on a rotating platform, at 4°C. For cell lysis, 100 μl of lysis buffer was added into each well of a 6-well plate. Crude cell lysate was cleared by centrifugation at 13,000 rpm, 4°C for 20 minutes. GST fusion proteins were immobilized on magnetic glutathione (GSH)-linked beads (Promega) by incubating 200 μl of the respective crude cell lysate with GSH-linked beads on a rotating platform for 30 minutes at room temperature. The beads were then washed 3 times with binding/washing buffer (Promega) and resuspended in 40 μl of the same binding/washing buffer. Cellular lysate (100 μl) from pHA-Rasd1 transfected cells was then incubated with the GST-Ear2 proteins immobilized on magnetic GSH-linked beads on a rotating platform for 1 hour, at room temperature, with 1% bovine serum albumin (BSA) as the blocking reagent. After incubation, the beads were vortexed once, and then washed five times with binding/washing buffer, and bound proteins were eluted from the GSH-linked beads by heating in Laemmli buffer at 95°C for 10 minutes.

In co-precipitation assays, COS-7 cells were co-transfected with pHisHA-Rasd1 and pGST-Ear2. Cells were harvested as described above. GST fusion proteins were immobilized on magnetic GSH-linked beads by incubating 200 μl of the cleared crude cell lysate with 30 μl of GSH-linked beads, with 1% BSA, on a rotating platform for 1 hour at room temperature. After incubation, the beads were washed four times with binding/washing buffer, and bound proteins were eluted from the GSH-linked beads by heating in Laemmli buffer at 95°C for 10 minutes.

Co-immunoprecipitations were performed by first pre-clearing 200 μl of crude cell lysate with 4 μg of mouse monoclonal IgG_1 _antibody (anti-c-Myc antibody, Santa Cruz), for 1 hour, on ice. The cell lysate was then incubated with 20 μl of protein G agarose resin beads (Invitrogen), for 30 minutes at 4°C on a rotating platform, followed by centrifugation at 14,000 × g for 10 minutes at 4°C. The supernatant was incubated with 4 μg of anti-GST antibody overnight, followed by 20 μl of resin beads for 1.5 hours, both on a rotating platform at 4°C. The resin was recovered after centrifugation at 13,000 rpm for 10 minutes at 4°C, washed once with PBS buffer and bound proteins were finally eluted from the resin beads by heating in Laemmli buffer at 95°C for 10 minutes.

Immunoprecipitation of endogenous Rasd1-Ear2 complexes was performed using crude lysate from untransfected HEK293T cells, as well as mouse brain crude lysate. Procedure was similar to co-immunoprecipitation protocol except that the crude cell lysate was pre-cleared with 80 μl of protein-G agarose resin beads for 30 minutes at 4°C. Goat polyclonal IgG anti-Ear2 antibody (Santa Cruz) was used to carry out immunoprecipitation, and a non-relevant goat polyclonal IgG anti-Tdg antibody (Santa Cruz) served as negative control. Immobilization of HisHA-Rasd1 was carried out using Ni-NTA Magnetic agarose beads (Qiagen) according to manufacturer's protocol. The Ni-NTA magnetic beads target the 5'-polyHis tag present on our HisHA-Rasd1 construct.

Proteins were analyzed using Western blots. Protein samples mixed with 1X Laemmli buffer were heated at 95°C for 10 minutes, separated by 10% SDS-PAGE, then transferred to polyvinylidene fluoride (PVDF) membranes (Bio-Rad, CA, USA). Blots were blocked in 5% non-fat milk, overnight at 4°C. Primary antibodies used included anti-GST mouse monoclonal IgG_1 _(1:5000) (Santa Cruz), anti-HA mouse monoclonal IgG_2a _(1:250) (Santa Cruz), anti-Ear2 goat polyclonal IgG (1:250) (Santa Cruz) and goat polyclonal anti-Rasd1 (1:1000) (Abcam). Secondary antibodies used included sheep anti-mouse horseradish peroxidase (HRP)-linked IgG (1:5000) (Amersham, GE Healthcare, UK) and rabbit anti-goat HRP-linked IgG (Abcam). Detection was performed with Western blot ECL kit detection reagents (Amersham, GE Healthcare, UK). All washes in between incubations are with TBST (0.1% Tween-20), for three times of 10 minutes each, at room temperature.

*Immunofluorescence staining*- Transfected COS-7 cells were used for indirect immunofluorescence staining as described [36]. HisHA-Rasd1 was detected with anti-HA antibody and visualized with goat anti-mouse IgG AlexaFluor 568 (Invitrogen). GST-Ear2 was detected and visualized with anti-GST AlexaFluor 488 (Santa Cruz) by confocal microscopy (Zeiss). Nuclei were stained by 4',6-diamidino-2-phenylindole (Sigma).

*Luciferase reporter assay*- Luciferase assays were performed using a Luciferase Assay System (Promega) kit according to manufacturer's protocol. COS-7, Neuro2a or As4.1 cells were co-transfected with pSV-β-Galactosidase, p4.1-Luc and relevant amounts of Ear2 and Rasd1 constructs. Total DNA concentration was held constant with respective carrier plasmid DNA. Cells were treated with 1 μM all-trans retinoic acid at 24 hours post transfection where applicable. Cells were harvested in 1X reporter lysis buffer (Promega) at 48 hours post transfection. The firefly luciferase activity was measured by a 20/20^n ^Luminometer (Turner Biosystems). β-galactosidase (β-gal) levels were measured using β-Galactosidase Enzyme Assay System (Promega) according to manufacturer's protocol. Luciferase activity was normalized against β-gal activity. Basal activity of promoterless enhancerless pGL3-basic was set to 100. Relative luciferase activities for all constructs were obtained from dividing normalized values against values obtained from promoter-less pGL3-basic. At least 3 sets of triplicates were conducted for each experiment. For all luciferase reporter assays, statistical analyses were performed using ANOVA or unpaired t-tests. Error bars shown are standard deviations.

*Real-time RT-PCR*- Total RNA was harvested from As4.1 cells 24 hours or 48 hours post transfection with Trizol reagent (Invitrogen) according to the manufacturer's protocol. The reverse transcription reaction was performed using iScript cDNA synthesis kit (Bio-Rad). Real-time PCR was performed with iTaq SYBR green supermix (Bio-Rad). The cycling conditions are 95°C for 15 seconds, 60°C for 30 seconds (for renin and G3PDH) or 66.5°C for 30 seconds (for Rasd1), 72°C for 40 seconds, 40 cycles. The number of PCR cycles for semi-quantitative real time RT-PCR was optimized to ensure that the reactions were in the linear range of amplification (G3PDH for 15 cycles, renin for 19 cycles and Rasd1 for 21 cycles). The amount of transcribed cDNA was normalized to G3PDH expression with the 7500 Real-time PCR system (Applied Biosystem). Three independent experiments were performed in duplicate.

Refer to Additional file 1 for oligonucleotides used in this study.

## Authors' contributions

JJT was involved in the design and execution of all experiments, and drafted the manuscript. SAO was involved in the knockdown and confocal studies, and edited the manuscript. K-SC conceived of the study, and participated in its design and coordination and edited the manuscript. All authors read and approved the final manuscript.

## Supplementary Material

Additional File 1 **List of oligonucleotides used for cloning and sequencing**. Tabulated data of oligonucleotides used in the experiments Click here for file
