# Supplementary material for: Rasd1 interacts with Ear2 (Nr2f6) to regulate renin transcription
Source: BMC Mol Biol. 2011 Jan 19;12:4. doi: 10.1186/1471-2199-12-4 (PMC3036621; doi:10.1186/1471-2199-12-4)
Supplement: Additional File 1 — List of oligonucleotides used for cloning and sequencing. Tabulated data of oligonucleotides used in the experiments [file 1471-2199-12-4-S1.DOC]

**Additional file 1: List of oligonucleotides used for cloning and sequencing**

| Primers used in yeast two-hybrid cloning and sequencing | | |
| --- | --- | --- |
| Primer name | Sequence | Description |
| Rasd1 F | 5'- cga*catatg*tatgaaactggccgcgatg -3' | Forward primer for PCR of Rasd1 (*Nde*I) |
| Rasd1 R | 5'- ggc*gaattc*actgatgacacagcgctcc -3' | Reverse primer for Rasd1 PCR (*Eco*RI) |
| MATCH MAKER 5' AD LD-Insert screening amplimer | 5'- ctattcgatgatgaagataccccaccaaaccc-3' | Forward primer for screening positive clones |
| MATCH MAKER 3' AD LD-Insert screening amplimer | 5'-gtgaacttgcggggtttttcagtatctacgat-3' | Reverse primer for screening positive clones |
| GAL4 AD sequencing primer | 5'- gggtttggaatcactacaggggta -3' | Sequencing primer for identifying positive clones |
| Primers used in the generating wild-type and mutant Rasd1 | | |
| HisHA-Rasd1 F | 5'-cga*ggtacc*tatgaaactggccgcgatg-3' | Forward primer for PCR of Rasd1 (*Kpn*I) |
| HisHA-Rasd1 R | 5'-ggc*gaattc*agcgtagtctgggacgtcgtatgggtaactgatgacacagcg-3' | Reverse primer for PCR of Rasd1 (*Eco*RI) |
| Rasd1[CAAX] F | 5'-cga*ggtacc*tatgaaactggccgcgatg-3' | Forward primer for generating Rasd1[CAAX] (*Kpn*I) |
| Rasd1[CAAX] R | 5'-ggc*gaattc*agcgtagtctgggacgtcgtatgggtagcgctccttgtccttagc-3' | Reverse primer for generating Rasd1[CAAX] (*Eco*RI) |
| Rasd1 flank F | 5'-cga*ggtacc*tatgaaactggccgcgatg-3' | Forward flanking primer for generating Rasd1 mutants (*Kpn*I) |
| Rasd1 flank R | 5'-ggc*gaattc*agcgtagtctgggacgtcgtatgggtaactgatgacacagcg-3' | Reverse flanking primer for generating Rasd1 mutants (*Eco*RI) |
| Rasd1[A178V] F | 5'-tcgagatctca**gtc**aagaagaacag-3' | Forward mutagenic primer for generating Rasd1[A178V] |
| Rasd1[A178V] R | 5'-ctgttcttctt**gac**tgagatctcga-3' | Reverse mutagenic primer for generating Rasd1[A178V] |
| Rasd1[G81A] F | 5'-tggacacatcc**gcc**aatcatccgttt-3' | Forward mutagenic primer for generating Rasd1[G81A] |
| Rasd1[G81A] R | 5'-aaacggatgatt**ggc**ggatgtgtcca-3' | Reverse mutagenic primer for generating Rasd1[G81A] |
| Rasd1[T38N] F | 5'-aagtgggcaag**aac**gccattgtgtc-3' | Forward mutagenic primer for generating Rasd1[T38N] |
| Rasd1 [T38N] R | 5'-gacacaatggc**gtt**cttgcccactt-3' | Reverse mutagenic primer for generating Rasd1[T38N] |
| Primers used in generating the full-length and truncated Ear2 | | |
| Ear2 F | 5'-cttctcgag*gcggccgc*tatggccatggtgaccggt-3' | Forward primer for PCR of Ear2 (*Not*I) |
| Ear2 R | 5'-cagcccggg*gcggccgc*tccagatacccatgacacca-3' | Reverse primer for PCR of Ear2 (*Not*I) |
| Ear2 N193 F | 5'-cttctcgag*gcggccgc*tatggccatggtgaccggt-3' | Forward primer for PCR of Ear2 N193 (*Not*I) |
| Ear2 N193 R | 5'-cagcccggg*gcggccgc*caactcgcacacgttgt-3' | Reverse primer for PCR of Ear2 N193 (*Not*I) |
| Ear2 N130 F | 5'-cttctcgag*gcggccgc*tatggccatggtgaccggt-3' | Forward primer for PCR of Ear2 N130 (*Not*I) |
| Ear2 N130 R | 5'-cagcccggg*gcggccgc*gcctcgctgcacggcctcct-3' | Reverse primer for PCR of Ear2 N130 (*Not*I) |
| Ear2 N53 F | 5'-cttctcgag*gcggccgc*tatggccatggtgaccggt-3' | Forward primer for PCR of Ear2 N53 (*Not*I) |
| Ear2 N53 R | 5'-gagcccggg*gcggccgc*caaccccggacgctcctcgt-3' | Reverse primer for PCR of Ear2 N53 (*Not*I) |
| Ear2 C54 F | 5'-cttctcgag*gcggccgc*gcaggtggactgcgtggtgt-3' | Forward primer for PCR of Ear2 C54 (*Not*I) |
| Ear2 C54 R | 5'-cagcccggg*gcggccgc*tccagatacccatgacacca-3' | Reverse primer for PCR of Ear2 C54 (*Not*I) |
| Ear2 C131 F | 5'-cttctcgag*gcggccgc*ccgcatcccgcatgagcgcc-3' | Forward primer for PCR of Ear2 C131 (*Not*I) |
| Ear2 C131 R | 5'-cagcccggg*gcggccgc*tccagatacccatgacacca-3' | Reverse primer for PCR of Ear2 C131 (*Not*I) |
| Ear2 C194 F | 5'-cttctcgag*gcggccgc*ggcacgcctgctgttcagca-3' | Forward primer for PCR of Ear2 C194 (*Not*I) |
| Ear2 C194 R | 5'-cagcccggg*gcggccgc*tccagatacccatgacacca-3' | Reverse primer for PCR of Ear2 C194 (*Not*I) |
| Primers used in generating the luciferase reporter construct | | |
| 4.1 F | 5'-gga*gctagc*agccctcttctggcctct-3' | Forward primer for PCR of 4.1kb of renin 5'-flanking sequences (*Nhe*I) |
| 4.1 R | 5'-aaa*aagctt*tagcccagaccccctgag-3' | Reverse primer for PCR of 4.1kb of renin 5'-flanking sequences (*Hind*III) |
| Oligonucleotides used in generating the shRNA knockdown constructs | | |
| Rasd1 shRNA F | 5'-*gatc*ccctgaaactggccgcgatgatttcaagagaatcatcgcggccagtttcattttta-3' | Oilgonucleotide sequences for generating Rasd1 shRNA (*Bgl*II) |
| Rasd1 shRNA R | 5'-*agctt*aaaaatgaaactggccgcgatgattctcttgaaatcatcgcggccagtttcaggg-3' | Oilgonucleotide sequences for generating Rasd1 shRNA (*Hind*III) |
| Control shRNA F | 5'-*gatc*cccgtcgaacggattgcacgtattcaaagagatacgtgcaatccgttcgacttttta-3' | Oilgonucleotide sequences for generating control shRNA (*Bgl*II) |
| Control shRNA R | 5'-*agctt*aaaaagtcgaacggattgcacgtatctcttgaatacgtgcaatccgttcgacggg-3' | Oilgonucleotide sequences for generating control shRNA (*Hind*III) |
| Primer used in real-time RT-PCR | | |
| Renin F | 5'-aggtttcctcagccaggactcgg-3' | Forward primer against renin mRNA |
| Renin R | 5'-ggccctgcctcccaggtcaa-3' | Reverse primer against renin mRNA |
| Rasd1 F | 5'-cgatccgcggcgaagtctac-3' | Forward primer against Rasd1 mRNA |
| Rasd1 R | 5'-gcggtgcaagtcggggctcatct-3' | Reverse primer against Rasd1 mRNA |
| G3PDH F | 5'-catccactggtgctgccaaggctgt-3' | Forward primer against G3PDH mRNA |
| G3PDH R | 5'- acaacctggtcctcagtgtagccca-3' | Reverse primer against G3PDH mRNA |
| Restriction sites are in italics, sequences for HA tag is underlined and bold font indicates mutated nucleotides. | | |
